# Health and demographic surveillance systems in low- and middle-income countries: history, state of the art and future prospects

**DOI:** 10.1080/16549716.2021.1974676

**Published:** 2022-04-04

**Authors:** Kobus Herbst, Sanjay Juvekar, Momodou Jasseh, Yemane Berhane, Nguyen Thi Kim Chuc, Janet Seeley, Osman Sankoh, Samuel J. Clark, Mark A. Collinson

**Affiliations:** a DSI-MRC South African Population Infrastructure Network, Durban, South Africa; b Population Science, Africa Health Research Institute, Durban, KwaZulu-Natal, South Africa; c KEM Hospital Research Centre, Vadu Rural Health Program, Pune, India; d Medical Research Council Unit, The Gambia at London School of Hygiene and Tropical Medicine, Fajara, The Gambia; e Addis Continental Institute of Public Health, Addis Ababa, Ethiopia; f Family Medicine Department, Hanoi Medical University, Hanoi, Vietnam; g Department of Global Health and Development, London School of Hygiene and Tropical Medicine, London, UK; h Statistics Sierra Leone, Tower Hill, Freetown, Sierra Leone; i Njala University, University Secretariat, Njala, Sierra Leone; j School of Public Health, Faculty of Health Sciences, University of the Witwatersrand, Johannesburg, South Africa; k Heidelberg Institute of Global Health, University of Heidelberg Medical School, Heidelberg, Germany; l Department of Sociology, The Ohio State University, Columbus, Ohio, USA; m SAMRC/Wits Rural Public Health and Health Transitions Research Unit (Agincourt), School of Public Health, Faculty of Health Sciences, University of the Witwatersrand, South Africa

**Keywords:** HDSS, Demography, Longitudinal Population Studies

## Abstract

Health and Demographic Surveillance Systems (HDSS) have been developed in several low- and middle-income countries (LMICs) in Africa and Asia. This paper reviews their history, state of the art and future potential and highlights substantial areas of contribution by the late Professor Peter Byass.

Historically, HDSS appeared in the second half of the twentieth century, responding to a dearth of accurate population data in poorly resourced settings to contextualise the study of interventions to improve health and well-being. The progress of the development of this network is described starting with Pholela, and progressing through Gwembe, Balabgarh, Niakhar, Matlab, Navrongo, Agincourt, Farafenni, and Butajira, and the emergence of the INDEPTH Network in the early 1990’s

The paper describes the HDSS methodology, data, strengths, and limitations. The strengths are particularly their temporal coverage, detail, dense linkage, and the fact that they exist in chronically under-documented populations in LMICs where HDSS sites operate. The main limitations are generalisability to a national population and a potential Hawthorne effect, whereby the project itself may have changed characteristics of the population.

The future will include advances in HDSS data harmonisation, accessibility, and protection. Key applications of the data are to validate and assess bias in other datasets. A strong collaboration between a national HDSS network and the national statistics office is modelled in South Africa and Sierra Leone, and it is possible that other low- to middle-income countries will see the benefit and take this approach.

## Background

This paper reviews the history, current state of the art and future of Health and Demographic Surveillance Systems (HDSS) in low- and middle-income countries (LMICs). These systems have been used to accurately account for a population at risk of exposure or subject to a particular intervention. The population is clearly defined within geographical boundaries and regularly monitored to document all in- and out-flows of people to and from the population.

Historically, HDSS appeared in the second half of the twentieth century, responding to a dearth of accurate population data in resource-challenged settings to contextualise the study of interventions to improve health and well-being. Early HDSS had much in common with the community-oriented primary care movement and provided evidence for interventions that became the mainstay of primary health care, such as oral rehydration for diarrheal treatment, bed nets for malaria prevention, and childhood vaccination.

Towards the end of the twentieth and early twenty-first centuries, HDSS was organised into networks with standardised methodologies and data structures, primarily facilitated through the INDEPTH Network. Over the last twenty years HDSS has contributed substantially as a research infrastructure to host a broad range of research studies and to boost research capacity building in LMICs. Longitudinal population cohorts organised into large international and regional consortia are gaining support from international funders and HDSS are capitalising on these developments to rekindle the work started by the INDEPTH Network.

The need for HDSS will continue given the dynamic nature of the health and socio-economic wellbeing in the face of global health challenges such as pandemics and climate change.

## History

### Roots

An important precept underlying the establishment of HDSS is that studying the cultural, socio-economic, and demographic characteristics of a population are fundamental to the understanding of, and intervening to improve, the health, and well-being of communities. In this perspective, HDSS have much in common with the community-oriented primary care movement [1,2] and Tollman links the development of the Agincourt HDSS (South Africa) to this movement [3]. Reviewing the establishment of the earliest demographic surveillance sites, this precept is evident in:

- Gwembe HDSS (Zambia), established in 1956 [4], to study the impact of the creation of Lake Kariba and associated resettlement of communities, the emphasis was on social and socio-economic issues and less on health [5].
- Ballabgarh HDSS (India), established in 1961 [6], to demonstrate a model health-care delivery system for rural India; its emphasis was on orientation and training medical students in primary health care.
- Niakhar HDSS (Senegal) established in 1962, the original emphasis was to demonstrate the recording of reliable demographic and epidemiological data in rural areas of Africa [7]. In later years Niakhar hosted important clinical vaccine trials for measles and pertussis, resulting in important changes to vaccination policies [8,9].
- Matlab HDSS (Bangladesh), established in 1966, also demonstrates this link between obtaining reliable demographic data and subsequent evaluation of interventions, in this case against diarrhoeal diseases [10]; but also added the explicit aim to act as a field site for the training of implementors of national health programmes. This objective is echoed in the Navrongo (Ghana) HDSS, established in 1993 in collaboration with the local health service [11–13].
- Farafenni HDSS (The Gambia), established in 1981, to evaluate a village-based primary health-care programme but has become an important site for malaria research [14].
- Butajira HDSS (Ethiopia), established in 1986, had a remit to establish an epidemiology research laboratory and develop local capacity in the prevention and control of disease and was closely associated with Addis Ababa University [15]. Butajira illustrates the important role of HDSS in building research capacity in LMICs.

The 1990s saw the establishment of several more HDSS throughout Africa: in Tanzania [16–18], South Africa [19,20], Burkina Faso [21], Mozambique [22], Uganda [23–25], Kenya [26], Malawi [27]; and also in South-East Asia [28,29].

### INDEPTH network

Established in 1998 [30] the INDEPTH Network (**I**nternational **N**etwork of field sites with continuous **D**emographic **E**valuation of **P**opulations and **T**heir **H**ealth) had a seminal impact on standardising HDSS methodology and data. The research sites initially focused on demographic and mortality monitoring, hence known as Demographic Surveillance System (DSS) sites. Their potential in evaluating the health status of the research participants was recognised and the DSS terminology was changed in 2008 to Health and Demographic Surveillance System (HDSS) sites. In fact, in 2015 the proposal to include morbidity monitoring as a core aspect of INDEPTH’s work led to the concept of Comprehensive Health and Epidemiological Surveillance System (CHESS) [31] (The acronym was coined by Professor Peter Byass). By 2017, the INDEPTH Network counted almost 50 HDSS member sites. The growing importance of the Network and its data were comprehensively described by Sankoh and Byass [32]. A later review article [33] highlighted the contributions of HDSS to science and development, research capacity building in LMICs and their role as a forum for researchers from LMICs to have a greater say in the research agenda conducted in LMICs. Since 2018, the INDEPTH Network’s influence waned due to disputes about its governance, and its disappearance from view contributed to the move to set up an African Population Cohorts Consortium (APCC) as discussed later.

The INDEPTH Network spawned several HDSS sub-networks and collaborations, specialising in specific topics, e.g. MADIMAH [34] on migration, ALPHA network [35] on HIV, on perinatal mortality [36], on non-specific effects of vaccines [37], on cause-specific mortality [38], on ageing [39], on non-communicable diseases [40], and on human genetics [41].

## State of the art

### HDSS methodology

The fundamental motivation for HDSS is the need to accurately account for the full population at risk of an exposure or subjected to a particular intervention, such as a clinical trial. With that in mind, a population is clearly defined and regularly monitored and all the in/out flows of people to/from the population are fully documented. The population is typically defined as everyone living within a geographical region; and the flows are birth and in-migration for the `ins’, and death and out-migration for the `outs’.

After an initial census, each household within the demographic surveillance area (DSA) is visited at least annually, and a detailed set of information on each household member is updated. Most HDSS sites conduct so-called `update’ rounds on either a quarterly, 3-monthly, half-yearly or yearly basis, largely dependent on funding considerations. This provides frequent updates on individuals, the households they live in and the community. The obvious balance that must be struck is between round frequency and cost, and the key factors that drive that balance are how well the site wants to characterize pregnancy-related outcomes and early child death, especially miscarriages, still births and neonatal mortality [42]. To do this well, the round frequency needs to be short enough to identify all pregnancies and their outcomes.

This basic design creates an observational platform capable of extremely intensive monitoring with respect to time, space, and a wide variety of social/health dimensions. The HDSS data are prospective, densely linked and very detailed. This provides the opportunity for simultaneous desegregation across many dimensions, and more unusual and useful, potential study of cause and effect [43] because the same entities (people, households, and communities) are followed through time. Although the details vary widely from site to site, an HDSS typically includes data on basic demography, socio-economic status (SES) through household asset information, cause of death (COD) through verbal autopsy (VA), various biomarkers and a wide variety of other social and biomedical data.

HDSS data are likely to be the timeliest and most detailed of all population/health data regularly generated in LMICs, and the HDSS platform is often used to conduct rigorous randomized controlled trials [44–48] and less rigorous observational studies of cause and effect [49–51]. The accumulated population and health data generated by HDSS sites are often used to conduct detailed retrospective population-based studies that include both sexes, all ages, all SES levels, etc. [52]. Data like these are usually extremely rare in LMICs.

### Data

The complexity of the data required to accurately record health and demographic surveillance data was recognised early [53], and the Household Registration System (HRS) [54] and its subsequent incarnations, HRS-2 [55] and OpenHDS [56] have been widely used by HDSS. Additionally, several data models [57–59] have been developed to represent the longitudinal nature of HDSS data in a more generic or abstract manner, beyond the confines of a specific software system. The INDEPTH Network published a standard data model [32] and data in this format from 29 different HDSS have been shared regularly on the INDEPTH Data Repository [60]. The MADIMAH collaboration developed a training manual to analyse data in this event history format [61].

### Strengths and limitations

The main strengths of HDSS data are their temporal coverage, detail, dense linkage, and the fact that they exist at all for the chronically under-documented populations in LMICs where HDSS sites operate. These data offer new perspectives because the level of precision is daily for births and in-migrations coming into the population, and deaths, by cause and for the out-migration of people leaving the population. With the population equation thus monitored, this means that for any given day the population membership can be reproduced and analysed.

The age-sex profiles say a lot about the population. In an example of a triangulation, where the age-sex profiles of the national population showed a seeming anomaly which was an expansion of the number of children under 5 years old, despite fertility steadily declining due to the increasing cost of child rearing. The HDSS showed the year-by-year transition over a fifteen-year period, which showed how the expansion in the number of children was a ripple effect caused by the youth bulge maturing and reaching their own fertile ages [62].

Time episodes are recorded of each person’s residency in a study village dwelling-place, as well as membership in a social unit, namely, a household. Non-residential household membership is also carefully recorded. This means that the HDSS platform can distinguish between permanent and temporary migration in the ongoing mobility surveillance. With dates of birth, death, in- and out-migration captured, after the initial baseline census, the population dynamics are tracked on a day-by-day basis. Models of continuous time event history analysis can be done with any of the constituent demographic variables − births, death, in and out-migration [61].

This level of intensity and detail also creates challenges associated with HDSS. The HDSS `study design’ is a 100% census of a geographically defined population and therefore does not represent any larger population in the sense of a sample survey. Consequently, results produced from HDSS data cannot be generalized to larger populations, although it is very tempting to do so! This lack of a statistical design that guarantees generalizability is one of the two very significant challenges for HDSS data. Byass [63] has shown that small area data at the scale of a HDSS can be closely indicative of national-level data; and Utazi [64] came to similar conclusions in the case of the drivers of childhood mortality, with certain exceptions.

The second limitation is the Hawthorne Effect [65,66]. HDSS study communities are observed comprehensively for long periods of time and participate in multiple, often overlapping, trials explicitly aimed at changing their health or behaviour. Even if adequate control groups are maintained for a study, those people are involved in other studies, and over the course of decades of being studied, it is certain that there is no unaffected control group, if that were even ethically feasible. Conversely, by only observing and not intervening to address adverse population health findings, it will expose the HDSS to an ethical dilemma [67,68].

The long-term engagement with populations in HDSS does raise ethical challenges because of the burden for participants of repeated rounds of data collection, ancillary care responsibilities and the expectations of local and direct benefits to individuals and communities linked to long-term engagement [69,70]. In recent years there has been a heightened awareness of the importance of investment in community engagement and the need for attention to the costs and benefits of data collection to participants as well as data access and use [67,71].

## The future

The 2030 Agenda [72,73] broadly calls for disaggregated population and health indicators describing national populations with frequent updates. In its current form, the HDSS method is suitable for this except that it does so for sub-national populations. It is therefore worth identifying what lessons can be learned and scaled-up from the HDSS; and how existing HDSS sites may contribute to the production of nationwide data.

HDSS is designed to study cause and effect relationships, and there will be a continuing need to conduct trials of all sorts. HDSS sites should continue doing this and should be expanded and replicated to provide LMICs with additional capacity to test pharmaceuticals, vaccines, and behavioural interventions locally; as well as monitor the impact of climate change on health, and the prospective documentation of the dynamics of risk factors for non-communicable diseases.

Although many LMICs do not have effective vital statistics and economic monitoring systems, they do have a variety of sources of data that can be combined to provide a reasonable description of the population and its health over time. Traditional data sources include the census, a wide variety of household surveys, some economic activity surveys, administrative records, facility-based (especially health) records, HDSS and other more ad hoc sources. Among these, HDSS data are usually the most detailed and the most accurate, but they suffer from the fact that they describe relatively small, geographically circumscribed populations. Combining data from multiple sources in many cases fills in temporal gaps in individual sources and covers much larger physical spaces. Examples of such triangulations exploiting detailed HDSS data from South Africa, include household definition [74], migration and settlement change [75] and civil registration and vital statistics [76,77].

One of the most exciting things to emerge in recent years is the potential of `big data’ to greatly improve the coverage, both spatial and temporal, and the content of routine data describing populations and their health. By definition, and in stark contrast to traditional data sources, big data do not have a statistical design that dictates how they are related to the population of interest, and therefore what they mean with respect to that population and how much variability they are expected to have. Traditional data sources are ‘samples’ of some kind, approaching 100% for the census or an HDSS, and much less for household surveys. In those cases, the resulting indicator values are generalizable to the population from which the sample was drawn, and uncertainty is largely related to sampling variability. Big data of the type that could or would not be used for population and health indicator production are all a by-product of other activities that have no statistical design whatsoever, so-called `digital exhaust’ [78]. A good example of this is cell phone call metadata [79] (the numbers from and to which a call is made along with the times when the call starts and ends and the cell towers to which the participating phones were linked at the time), effectively a location for each phone. This is a lot of useful information, but it pertains to people who have cell phones and use them. Such data cannot say anything about people without cell phones, or those with phones who never use them. It is easy to see that data of this type are biased in potentially many ways! Perhaps the most valuable use of HDSS sites in the era of big data will be to characterize this bias in big data. By adding a detailed `cell phone module’ to ongoing HDSS data collection, it will be possible to understand cell phone ownership and usage in detail in addition to all the other information describing the HDSS study populations. Then, by combining all of this with cell phone call metadata that includes the cell phones used by the HDSS study population, it will be possible to characterize and understand the biases and omissions inherent in the cell phone call metadata, and that understanding can be used to de-bias, calibrate, and adjust indicator values produced using cell phone call metadata that describe large populations that are like the HDSS study population.

Although we have used cell phone call metadata as an example, this general approach should work for any type of big data, social network data, satellite imagery, and so on. This is not a new concept, ‘ground truthing’ has been practiced by geographers and mapmakers for a long time. In this case we are ground truthing big data by calibrating them using the fact that in the HDSS we can understand both the big data and the indicators we are interested in and relate them to one another.

Point of contact interactive record linkage [80,81] in HDSS coupled with advances in consolidating electronic medical records into integrated data warehouses [82] shows promise in enhancing measurement of universal health coverage.

Accessing, storing, and using data from HDSS will continue to pose ethical challenges and dilemmas as we move forward. Broad consent, where a participant consents to their data/samples being used for future research of certain types, has been used increasingly over the past decade [83]. However, critics argue that it can be misleading to ask participants for informed consent for research that is unforeseen and not specified [84]. Dynamic consent can address such concerns by setting out to obtain consent for every future research project using stored data [85]. Shifting public views and perceptions of the utilisation of stored data as well as other information (e.g. cell phone, social network, and genomic data) from HDSS as well as the opinions and guidance of national ethical review bodies and governments provides opportunities for researchers and HDSS participants to contribute to ongoing debates about research in Africa and beyond. Noteworthy is the fact that even though HDSS generates vital evidence for health researchers, academicians, policy and practice, sustainability of HDSS is always at stake [86,87]. Sustainability is a problem for many HDSS and greater recognition by national governments of the importance of the research infrastructure and capacity-building opportunities offered by HDSS translating into long-term funding, could address this [88,89].

### Networking and funding

In developing the use of scientific findings to provide multi-level evidence for policy making, there is much efficiency and mutual value gained in the development of national research infrastructures, as partnerships of government, universities, and research communities. The South African Population Research Infrastructure (SAPRIN) is such a network of longitudinal population and health surveillance system nodes [89], funded by the South African Department of Science and Innovation (DSI) as part of the South African Research Infrastructure Roadmap [90] (SARIR) to implement a network of interconnected national research infrastructures in different domains of science relevant for policymaking. As a national research infrastructure, SAPRIN receives long-term government funding and produces evidence in official reports and scientific articles, conducts data management and data sharing, and has ongoing engagement with government ministries involved in policymaking. It also supports capacity development and post-graduate research at South African and other universities.

Statistics Sierra Leone, the National Statistics Office (NSO) of Sierra Leone, has received funding from the World Bank to establish HDSS sites in Sierra Leone. This is the first time in the history of health and demographic surveillance for an NSO to be the centre running HDSS sites. Together with SAPRIN in South Africa it signals the continued potential for such systems to become embedded in the policymaking and research infrastructure in LMICs.

There has been an increasing focus on bringing together population cohorts into consortia to exploit their potential for harnessing large and diverse datasets for precision human health research [91–93] and an emphasis of the need to include African subjects in these studies [94]. The Wellcome Trust has recently published a report into the scope for an African Population Cohorts Consortium [95] and will be funding the formative phase of such a consortium.

## Conclusion

In the space between full coverage of vital statistics, an enviable state not yet achieved by most LMICs and being limited to the statistics available from providing public services lies the importance of HDSS. The recent growth of knowledge about HDSS and advances in the technology for it, along with increasing numbers of population cohorts in countries of the global South offers an opportunity to continue and enhance the contribution of longitudinal population cohorts to science and policy.

As has been described, there are several key stakeholders of HDSS nodes. The relationship between the researchers and the community under surveillance is fundamental to this work. The onus is on the researchers to provide feedback on useful information learned in the research and provide a fair warning of new work to be implemented. Consent and evidence of this have always been at the foreground at HDSS and the new standards set by information policies are welcomed. Another stakeholder is the broader community of researchers. Multi-disciplinary research embedded in cohorts and other methodologies with post-doctoral training in place are to be encouraged and facilitated. Government policy-makers are the third set of key stakeholders to recognise. They especially benefit from HDSS nodes when their data are harmonised and furthermore if the data are triangulated with national census data. The national statistical infrastructure can be hugely enhanced by HDSS nodes operating in the country.

Through this article the growing importance of health and demographic surveillance systems has been highlighted. This need for HDSS nodes will continue due to the dynamic nature of the health and socio-economic wellbeing in the face of global health challenges such as pandemics and climate change.

### Peter Byass role in the evolution of HDSS

**Peter Byass and the Farafenni HDSS: preparing the ground for computerized management of demographic surveillance data in rural West Africa**

When the pioneers of On-line Tropical Epidemiology in rural Gambia in the early 1980s realized the need to maintain a database management system that fulfills the demographic requirements of their studies, it coincided with the availability of two vital inputs – the introduction of microcomputers and the presence of Peter Byass. This came soon after a system for continuous demographic monitoring of a study population was set up in Farafenni, with annually updated questionnaires sent 180 km away to the UK's MRC Head Office in Fajara, The Gambia or to London, UK, for entry and processing. With the relational database management system (RDBMS) trending at the time and the rudimentary technology at his disposal, Peter went on to design the first generation of computer forms for both cross-sectional and long-term epidemiological studies in rural West Africa [96]. Using BBC microcomputers of barely 20 Mb capacity and dBase II, Peter and colleagues pulled together a demographic surveillance system with greatly improved speed and quality of data processing, as well as accessibility of data for epidemiological purposes [53]. Their efforts constituted the humble beginnings of the Farafenni HDSS; and the valuable experience Peter so deservedly shared to set up other HDSS sites in Eastern and Southern Africa, and south-east Asia.

**Peter Byass and the Butajira HDSS: supporting HDSS data management and research capacity building**

The Butajira HDSS was established in 1986. Initially, the data were collected on paper and the database used to handle such a complex relational data was rudimentary and prone to serious errors. Thus, reconciling the various vital registration systems (birth, death, and migration) and updating the main database was an arduous task. Peter joined the Butajira HDSS research group in 1994. Cognizant of the serious challenges the team faced in managing the database at that time, one of the Peter’s first contributions to the Butajira HDSS was the development of an in-house relational database that he called ‘Buta’. The Buta database was designed and written by Peter himself. He was also instrumental in shaping the data structure in a way that is suitable for longitudinal data analysis. Peter was also key to mentoring many young researchers from Ethiopia and Sweden in longitudinal data analysis. As a result, the data management and analysis capacity of the Butajira research team immensely improved and the scientific productivity of the group grew exponentially. Peter has supervised several Ethiopian PhD students who used the Butajira data in subsequent years. Peter’s ability to contextualize data analysis and interpretational skills have greatly benefitted his PhD students and policy dialogue based on evidence generated from Butajira HDSS. The Butajira DSS eventually moved its data management system to the Household registration System (HRS).

**Peter Byass and the Filabavi HDSS: continuing research capacity building and developing automated verbal autopsy interpretation**

In 1996, the Health Systems Research Project (HSRP), a collaboration between Vietnamese and Swedish public health scientists, identified the need for a field site to improve the availability of reliable health data for formulating and monitoring health system change and the FilaBavi HDSS, an epidemiological field laboratory sited in the Bavi District was established. Peter’s early influence at Filabavi is documented in a supplement to the Scandinavian Journal of Public Health [97] and through the published work of the PhD students he supervised there, which ranged from injuries [98], socioeconomic determinants of hypertension [99] and quality of life of the elderly [100]. It is here where he did his early work on verbal autopsies [101] and their automated interpretation [102], probably the achievement Peter is best known for.

## Data Availability

N/A

## Data deposition

N/A

## Paper context

Health and Demographic Surveillance Systems (HDSS) in low- and middle-income countries provide data for the study of the cultural, socio-economic, and demographic characteristics of a population, fundamental to the understanding of, and intervening to improve, the health and well-being of communities. The need for HDSS will continue due to the dynamic nature of the health and socio-economic wellbeing, particularly in the face of global health challenges such as pandemics and climate change.
